# Curcumin Activates AMPK Pathway and Regulates Lipid Metabolism in Rats Following Prolonged Clozapine Exposure

**DOI:** 10.3389/fnins.2017.00558

**Published:** 2017-10-04

**Authors:** Zhen Liu, Changmeng Cui, Pengfei Xu, Ruili Dang, Hualin Cai, Dehua Liao, Mengqi Yang, Qingyan Feng, Xin Yan, Pei Jiang

**Affiliations:** ^1^Department of Pharmacy, Affiliated Hospital of Jining Medical University, Jining, China; ^2^Institute of Clinical Pharmacy and Pharmacology, Jining First People's Hospital, Jining Medical University, Jining, China; ^3^Department of Pharmacy, Second Xiangya Hospital, Central South University, Changsha, China; ^4^Department of Pharmacy, Hunan Cancer Hospital, Central South University, Changsha, China; ^5^Research Center for Drug Discovery, School of Pharmaceutical Sciences, Sun Yat-sen University, Guangzhou, China

**Keywords:** curcumin, clozapine, AMPK, SREBP, dyslipidemia

## Abstract

Clozapine (CLO) remains an ultimate option for patients with treatment resistant schizophrenia. However, the atypical antipsychotic is often associated with serious metabolic side effects, such as dyslipidemia. Hepatic sterol regulatory element-binding proteins (SREBPs) are central in the allosteric control of a variety of lipid biosynthetic pathways. There is emerging evidence that CLO can activate SREBP pathway and enhance downstream lipogenesis, whereas curcumin (CUR), a major active compound of *Curcuma longa*, contains hypolipidemic properties. Therefore, in the present study, we examined the protective effects of CUR against CLO-induced lipid disturbance and analyzed the expression of key components in hepatic lipid metabolism. Our data showed that 4-week treatment of CLO (15 mg/kg/day) markedly elevated serum lipid levels and resulted in hepatic lipid accumulation, whereas co-treatment of CUR (80 mg/kg/day) alleviated the CLO-induced dyslipidemia. We further demonstrated that CUR appears to be a novel AMP-activated protein kinase (AMPK) agonist, which enhanced AMPK phosphorylation and mitigated CLO-induced SREBP overexpression. Additionally, CUR also modulated the downstream SREBP-targeted genes involved in fatty acid synthesis and cholesterol metabolism, including fatty acid synthase (FAS) and HMG-CoA reductase (HMGCR). In summary, our study suggests that the suppressed AMPK activity and thereby enhanced SREBP-dependent lipid synthesis could be associated with the antipsychotic-stimulated dyslipidemia, whereas CUR may maintain lipid homeostasis by directly binding to AMPK, indicating that adjunctive use of CUR could be a promising preventive strategy for the drug-induced lipogenesis.

## Introduction

High rates of comorbidity with metabolic syndrome are associated with schizophrenia patients following treatment with atypical antipsychotic drugs (AAPDs), such as clozapine (CLO) and olanzanpine (Kristóf et al., 2016). Although, CLO remains an ultimate option for patients with treatment resistant schizophrenia, it has the worst metabolic profile of all antipsychotics. The AAPD-induced dyslipidemia and other metabolic disorders are troublesome, which may eventually contribute to the increased cardiovascular mortality and premature death in this debilitating disease (Blanco et al., 2014). Therefore, tackling the causes of metabolic disease in schizophrenia is of paramount importance, and finding strategies to alleviate the metabolic effects of antipsychotics is a top priority in this regard.

The molecular mechanisms by which AAPDs drive such metabolic disturbances are intricate and remain poorly understood. Although, the drug-induced dyslipidemia might be secondary to hyperorexia and obesity, recent evidence highlights that the antipsychotics can also exert direct effects on lipid metabolism in peripheral tissues (McNamara et al., 2011). AMP-activated protein kinase (AMPK) has emerged as a key regulator in hepatic energy metabolism and lipid homeostasis. Activation of AMPK suppresses fatty acid and cholesterol biosynthesis, by inhibiting the activity of acetyl CoA carboxylase (ACC) and 3-hydroxy-3-methyl glutaryl CoA reductase (HMGR; Li et al., 2014; Choi et al., 2017). In addition, AMPK can inactivate sterol regulatory element-binding proteins (SREBPs), the major transcriptional regulators of lipogenesis, and thereby inhibit the downstream lipogenic genes (Jang et al., 2017). Recent studies demonstrated that the AAPDs perturbs AMPK signaling and activates SREBP signaling, resulting in the overexpression of downstream lipogenic genes and dyslipidemia, whereas pharmaceutical means to potentiate hepatic AMPK are promising to maintain lipid homeostasis during AAPD treatment (Oh et al., 2011).

Curcumin (CUR), a yellow coloring component of the rhizome of *Curcuma longa*, has caught much attention as an effective remedy for multiple disorders, such as dyslipidemia and hyperglycemia (Panahi et al., 2016). It has been demonstrated that CUR can rescue obesity and reduce lipogenesis in the high-fat diet mouse model through regulating SREBP pathway (Ding et al., 2016). Although the regulatory effects of CUR on SREBP-dependent lipid synthesis are proved by both *in vivo* and *in vitro* studies (Kang and Chen, 2009; Ding et al., 2016), the mechanisms by which CUR restores SREBP over activation are still elusive. Therefore, the main objective of the study was to evaluate the effects of CUR on CLO-induced metabolic abnormalities and AMPK-SREBP signaling was also examined to further elucidate the potential mechanisms.

## Materials and methods

### Animals

Male, Sprague–Dawley rats (200–230 g) were housed under standard conditions of temperature (23 ± 2°C) and light (12:12 h light/dark cycle), with free access to standard rodent chow (3% fat, 3.2 kcal/g) and water. Each rat was housed in a separate cage and recorded with body weight and food intake daily. All animal use procedures were carried out in accordance with the Regulations of Experimental Animal Administration issued by the State Committee of Science and Technology of the People's Republic of China, with the approval of the Ethics Committee in our university (NO.20160076).

### Experimental design

Rats were randomly allocated to one of the four groups (*n* = 9): control, CUR, CLO, and CLO+CUR. Rats in CLO and CLO+CUR group received CLO (Eastbang Pharmaceuticals, China) via subcutaneous injection at a dose of 15 mg/kg for 4 weeks. CLO was formulated in 0.9% saline containing 0.2% acetic acid and 0.5% Tween 80 and the dosing solutions were prepared fresh daily. The dose for the antipsychotic was chosen based on our previous researches (Cai et al., 2015, 2017; Dang et al., 2015), which was converted from clinically prescribed dosages using the body surface area normalization method, and we chose subcutaneous injection to avoid the significantly shorter half-life of CLO in rodents as compared with humans (Mann et al., 2013). CUR (Sigma-Aldrich, USA) was also suspended in 0.5% Tween 80 and administrated daily by oral gavage (100 mg/kg) about 1 h before CLO treatment. Dose and regimen of CUR treatment was selected based on previous findings showing its beneficial effects against streptozotocin-induced renal lipid accumulation (Soetikno et al., 2013). Vehicle (VEH) for subcutaneous injection consisted of 0.9% saline acidified with 0.2% acetic acid and 0.5% Tween 80. Body weight of these rats was monitored throughout the experiment, and the drug doses were adjusted accordingly. After the experiment, food was withdrawn from animals 12 h before sacrifice and trunk blood was collected. The liver median lobe tissues were rapidly dissected and freeze-clamped in liquid nitrogen before storage at −80°C. Perirenal and epididymal fat pads were dissected and weighed to provide an index of visceral adiposity. Visceral fat deposition was measured as a percentage of the total weight of perirenal fat and epididymal fat relative to final body weight.

### Biochemical analysis

Serum status of free fatty acids (FFAs) and total cholesterol (TC) were determined by enzymatic colorimetric assays using commercial kits (Sekisui Medic, Japan). Triglyceride (TG) was measured using the TG glycerol phosphate oxidase assay (Abbott Laboratories, USA) and Glucose was determined by the hexokinase method with a commercial kit (Abbott Laboratories, USA). Hepatic total cholesterol (TC) and triglyceride (TG) were extracted with a chloroform-methanol (2:1, vol/vol) mixture according to the Folch method and then were determined by colorimetric assay kits (Cayman Chemical, USA).

### Western blot analysis

For western blotting analysis, total protein was prepared from liver tissue, and the protein concentrations were analyzed using Bradford method. Samples were loaded on precast 12% SDS-PAGE gels with ~50 μg protein in each lane. The following antibodies and concentrations were used over night at 4°C; phosphor-AMPK (Thr172) (Cell Signaling; 1:2,000), AMPK (Cell Signaling; 1:2,000), SREBP-1 (Santa Cruz; 1:200), SREBP-2 (Santa Cruz; 1:500), and β-actin (Proteintech; 1:4,000). The signals were normalized to the housekeeping gene, β-actin, as an internal standard. Variations in the density were expressed as fold changes compared with the control in the blot.

### Real-time PCR analysis

Total RNA was extracted by using Trizol reagent (invitrogen, USA) following the manufacturer's instructions. Quantitative PCR was performed on Bio-rad Cx96 Detection System (Bio-rad, USA) using SYBR green PCR kit (Applied Bio-systems, USA) and gene-specific primers (Table 1). Each cDNA was tested in triplicate. Thermo profile conditions were: 50°C for 2 min, 95°C for 10 min, and 40 cycles of amplification at 95°C for 15 s and 60°C for 1 min. Relative quantitation for PCR product was normalized to β-actin as an internal standard.

**Table 1:** Primer sequences used for the qPCR analysis.

**Gene**	**Sense primer (5′-3′)**	**Antisense primer (5′-3′)**
ACC1	CAACGCCTTCACACCACCTT	TCATCAAAGATCCTGACGAAATCTT
FAS	CCATCATCCCCTTGATGAAGA	GTTGATGTCGATGCCTGTGAG
HMGCS	CAGCTCTTGGGATGGACGA	GGCGTTTCCTGAGGCATATATAG
HMGCR	TGGCCAGGATGCAGCAC	GGCATGGTACAGCTGATGTATAAGTC
LDLR	ACCGCCATGAGGTACGTAAG	ACCGCCATGAGGTACGTAAG
β-Actin	CATCCTGCGTCTGGACCTGG	TAATGTCACGCACGATTTCC

### Molecular modeling

The crystal structure of adenosine monophosphate-activated protein kinase (AMPK) was loaded from the Protein Data Bank (PDB ID: 5T5T) in the docking study. The molecular structure of CUR was optimized using MMFF94s force filed, and then AMPK was protonated based on amber99 force filed after removing water molecules. MOE-docking (Chemical Computing Group, Inc. Montreal, Canada) was applied to identify the binding poses of CUR and AMPK. All docked poses of CUR were ranked on the basis of the binding docking energies. The lowest energy conformation was chosen for binding modes analyses.

### Statistical analysis

All statistical procedures were performed on SPSS version 18. Data were expressed as mean ± SD. For body weight and food intake data, repeated measures analysis of variance (ANOVA) with drug treatment as the between subject factor and week or test day as the within subject factor were conducted. Visceral fat deposition and the biochemical data were analyzed by two-way ANOVA. When appropriate, Dunnett's test was used for post hoc multiple comparisons to determine significant difference between groups. The prior level of significance was established at *P* < 0.05.

## Results

### Body weight and food intake

**Figure 1:**
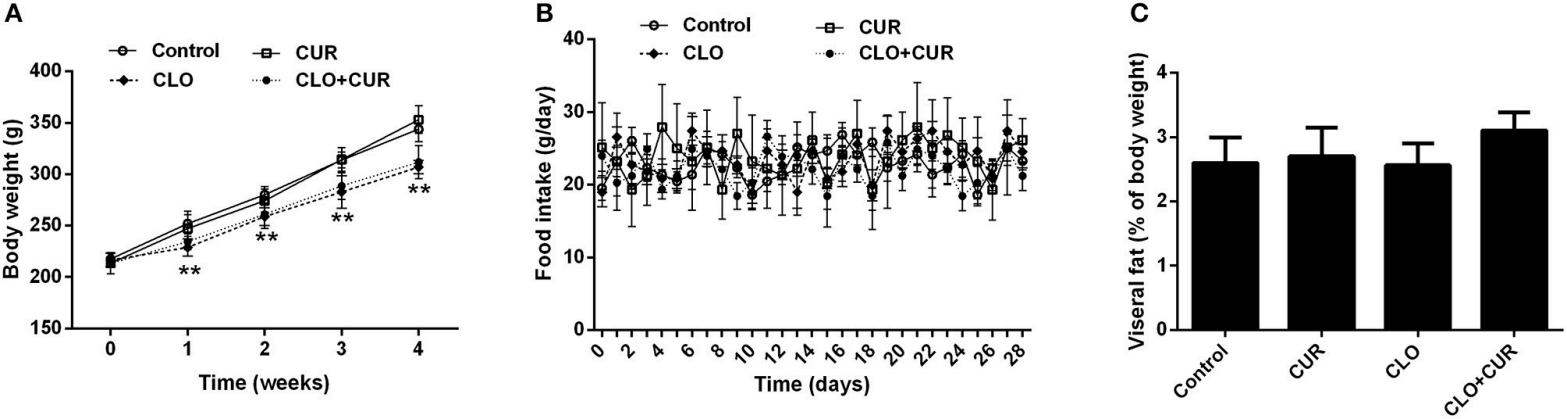
Effect of clozapine (CLO) and curcumin (CUR) treatment on body weight **(A)**, daily food intake **(B)**, and visceral fat deposition **(C)** in rats. Visceral fat deposition was measured as a percentage of the total weight of perirenal fat and epididymal fat relative to final body weight. Data are means ± SD. ^**^p < 0.01 compared to control group.

As shown in Figure 1, CLO suppressed the body weight gain [*F*_(3, 32)_ = 9.75, *p* < 0.01] from the first week to the end of the experiment without affecting daily food intake [*F*_(3, 32)_ = 2.83, *p* > 0.05], whereas CUR had no effect on the body weight growth (*p* > 0.05) and food consumption (*p* > 0.05) in both control and CLO-treated animals. In addition, neither CLO nor CUR treatment affected visceral fat deposition. The results were consistent with our previous findings (Cai et al., 2015; Dang et al., 2015), indicating that CLO-induced metabolic disturbance is independent of its effects on hypothalamic neurotransmission and consequent changes in food intake and obesity, but may through the direct modification on lipid metabolism in the periphery.

### Serum and hepatic levels of lipids

**Figure 2:**
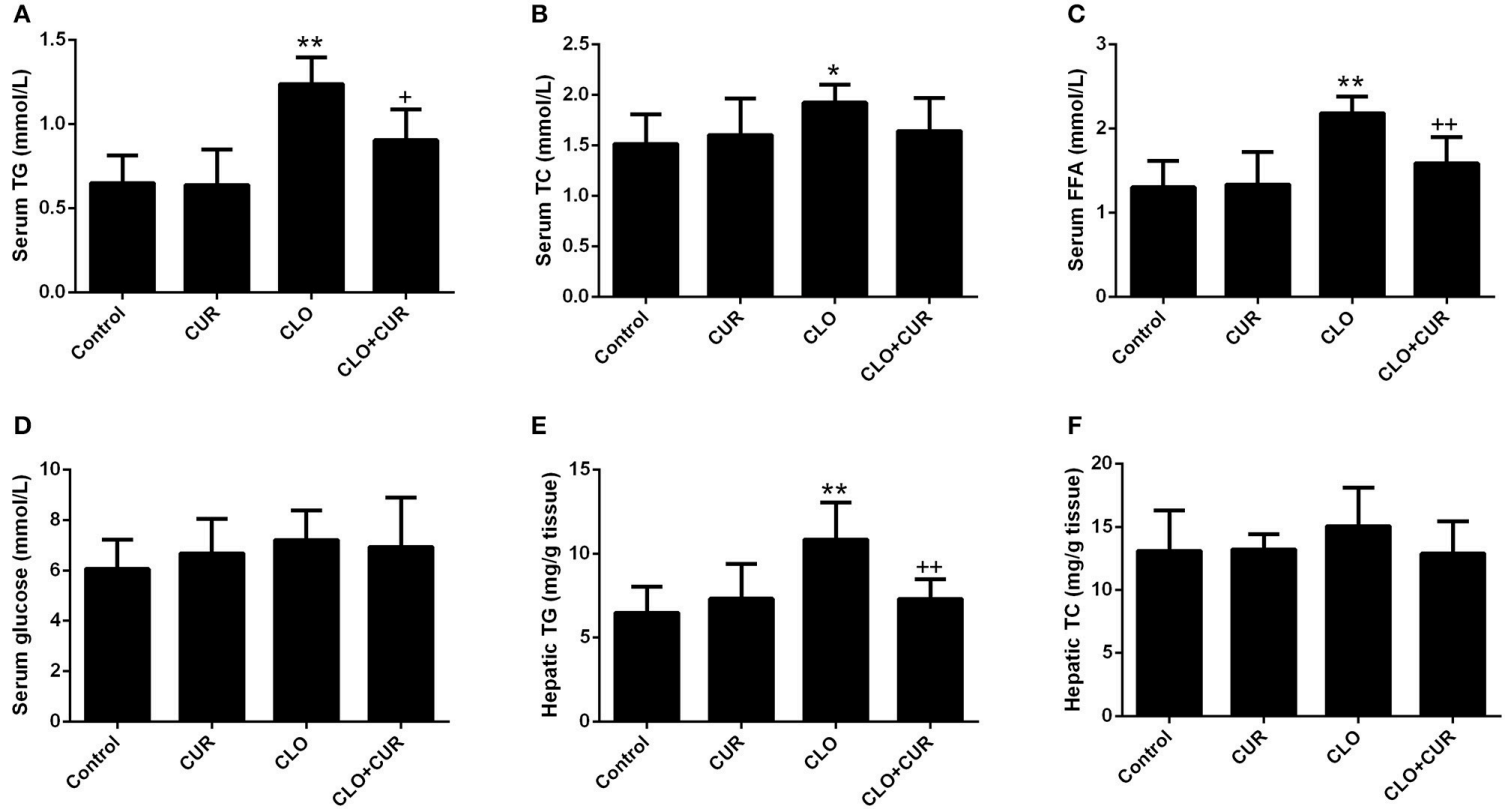
Effect of clozapine (CLO) and curcumin (CUR) treatment on serum concentrations of Triglyceride (TG) **(A)**, total cholesterol (TC) **(B)**, free fatty acids (FFAs) **(C)** and glucose **(D)**, and hepatic status of TG **(E)** and TC **(F)** in rats. Data are means ± SD. ^*^p < 0.05, ^**^p < 0.01 compared to control group. ^+^*p* < 0.05, ^++^*p* < 0.01 compared to CLO group.

As we previously reported (Cai et al., 2015), CLO perturbed lipid homeostasis with significant increase of serum concentrations of TG (Figure 2A; main effect of CLO, *F*_(1, 32)_ = 50.46, *p* < 0.01) and TC (Figure 2B; main effect of CLO, *F*_(1, 32)_ = 5.13, *p* < 0.05), whereas CUR attenuated the elevated TG status (CUR × CLO interaction, *F*_(1, 32)_ = 5.39, *p* < 0.05) and slightly, but nonsignificantly decreased TC levels in the CLO treated animals (CUR × CLO interaction, *F*_(1, 32)_ = 3.47, *p* = 0.07). CLO treatment also increased serum FFA concentration, which was mitigated by CUR administration (Figure 2C; *F*_(1, 32)_ = 11.57, *p* < 0.01). Serum glucose was unchanged in both CLO and CUR treated rats (Figure 2D). In parallel, CLO also significantly enhanced hepatic TG genesis (Figure 2E; *F*_(1, 32)_ = 13.22, *p* < 0.01), whereas CUR mitigated the CLO-induced elevation of hepatic TG status (*p* < 0.01). Additionally, neither CLO nor CUR altered TC concentration in the liver (Figure 2F).

### Effect of CUR and CLO on AMPK-SREBP signaling and lipogenetic genes

**Figure 3:**
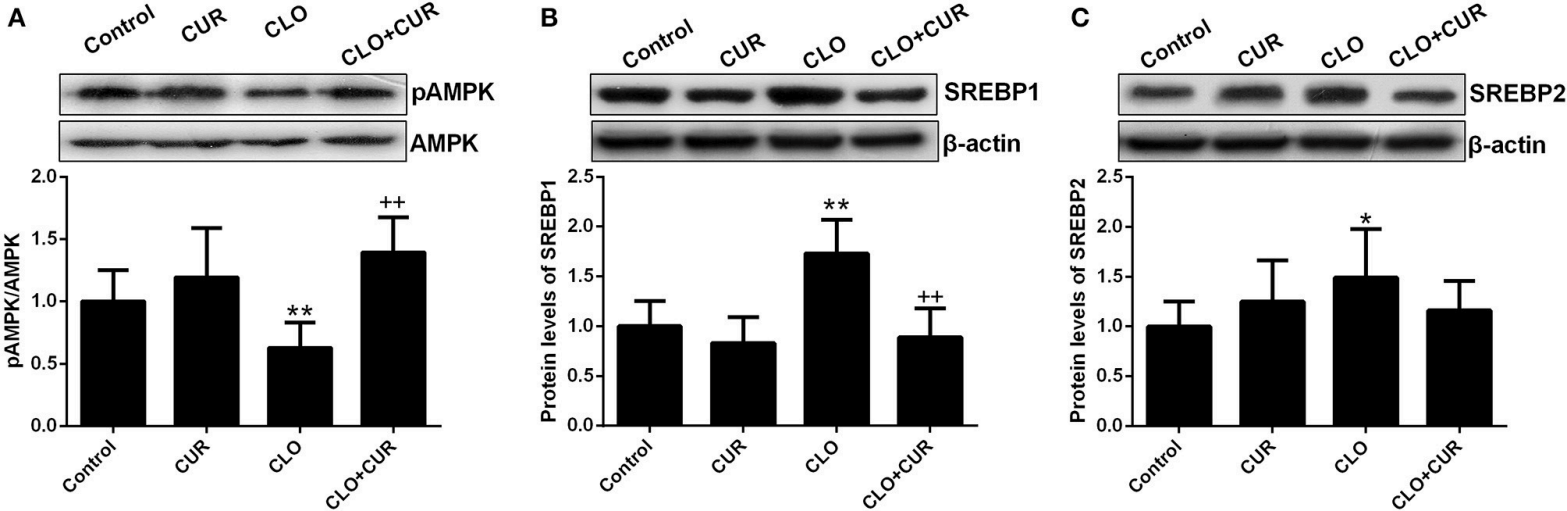
Effect of clozapine (CLO) and curcumin (CUR) treatment on AMPK phosphorylation **(A)** and the protein expression of SREBP-1 **(B)** and SREBP-2 **(C)** in rat liver. Data are means ± SD. ^*^p < 0.05, ^**^p < 0.01 compared to control group. ^++^*p* < 0.01 compared to CLO group.

**Figure 4:**
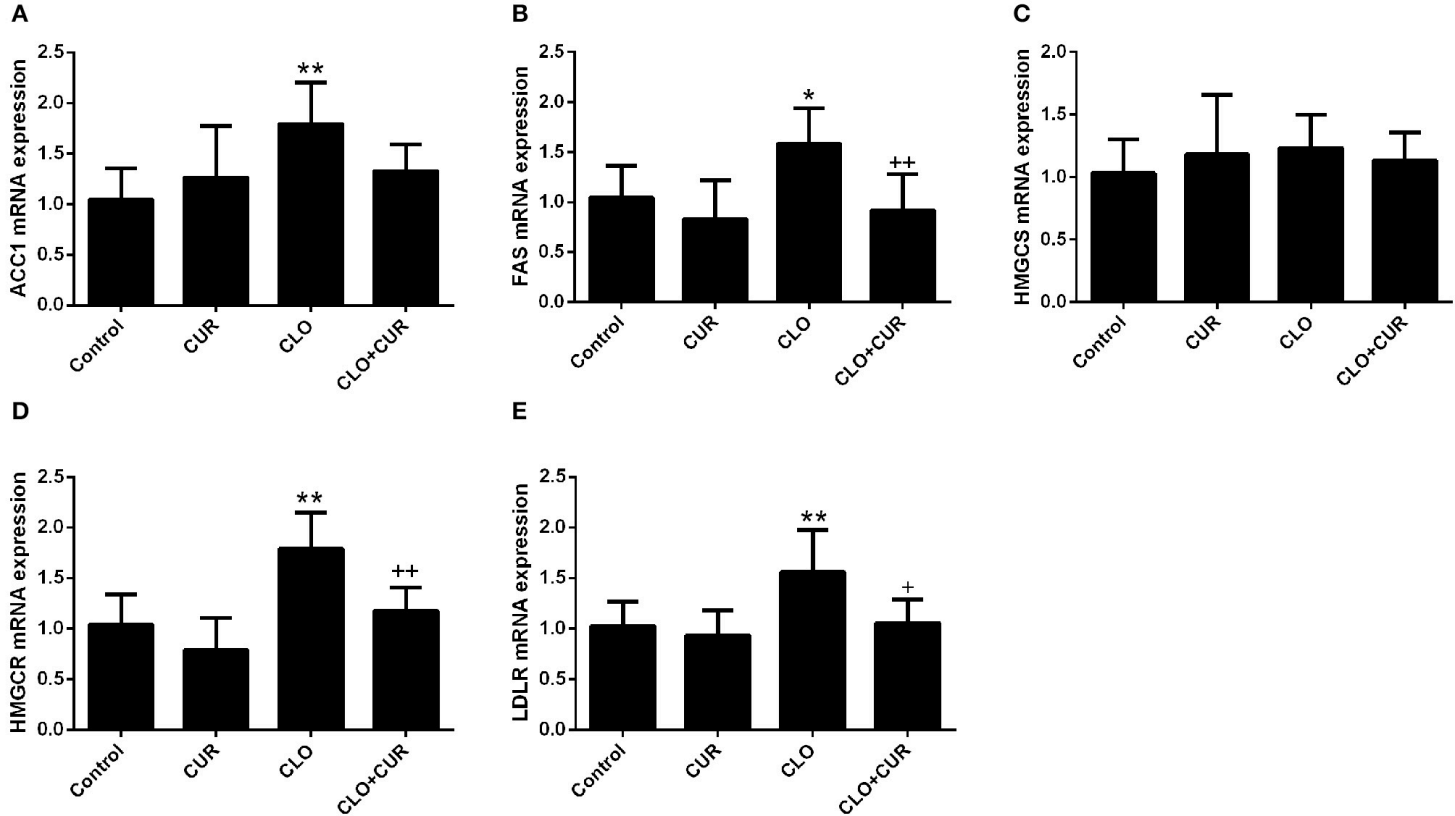
Effect of clozapine (CLO) and curcumin (CUR) treatment on the hepatic gene expression involved in fatty acid synthesis (ACC and FAS) **(A,B)** and cholesterol metabolism (HMGCS, HMGCR, and LDLR) **(C–E)**. Data are means ± SD. ^*^p < 0.05, ^**^p < 0.01 compared to control group. ^+^*p* < 0.05, ^++^*p* < 0.01 compared to CLO group.

To elucidate the underlying mechanisms by which CUR partly alleviated the antipsychotic-induced lipid disturbances, hepatic protein levels of the AMPK-SREBP pathway were analyzed. For p-AMPK/AMPK ratio, the ANOVA analysis revealed a main effect of CUR [*F*_(1, 32)_ = 24.16, *p* < 0.01] and CLO [*F*_(1, 32)_ = 9.13, *p* < 0.01], with a significant interaction between both factors [*F*_(1, 32)_ = 17.19, *p* < 0.01; Figure 3A]. Prolonged CLO administration reduced AMPK phosphorylation with profoundly decreased p-AMPK/AMPK ratio (*p* < 0.01) and markedly induced both SREBP-1 (*p* < 0.01) and SREBP-2 (*p* < 0.05) expression (Figures 3B,C). Meanwhile, co-administration of CUR enhanced AMPK activation and restored the overexpressed SREBP-1 and SREBP-2. Additionally, consistent with SREBP results, CUR also downregulated the CLO-induced overexpression of SREBP target genes involved in fatty acid and cholesterol synthesis, including ACC1, fatty acid synthetase (FAS), HMGR, and LDL-receptor (LDLR; Figure 4). These data suggest that CUR can mitigate CLO-induced dyslipidemia through activate AMPK.

### Molecular docking of CUR with AMPK

**Figure 5:**
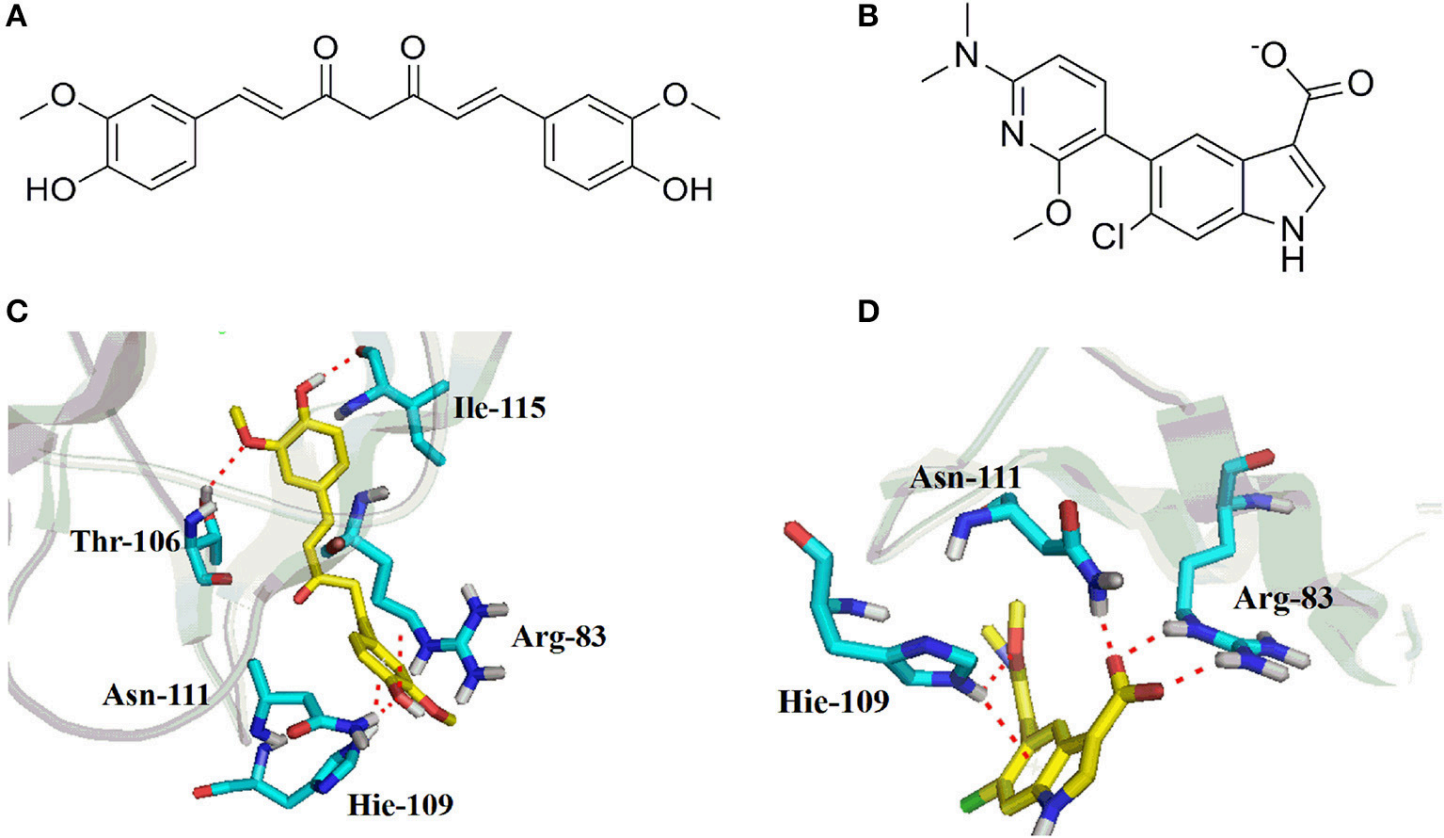
Chemical structures of curcumin (CUR) **(A)** and its reported native ligand, PF-249 **(B)**, and the binding property of CUR **(C)**, and PF-249 **(D)** with AMPK.

To further understand the binding property of CUR (Figure 5A) with AMPK protein, a molecular docking analysis was performed. As shown in Figure 5, the results from the computational docking model suggested that the hydroxyl groups of CUR can form three hydrogen bonds with the residue Arg83, Arg111, and Leu115, respectively, at the allosteric regulatory site of AMPK (PDB: 5T5T). Oxygen atom in methyl ether group of CUR and the residue Thr106 form a hydrogen bond together. In addition, the benzene ring of CUR forms π-π stacking interactions with the residue Hie109 (Figure 5C). The interaction can provide the binding energy to stabilize the CUR–AMPK complex. For more precious result, we further docking AMPK with its reported native ligand, 6-chloro-5-[6-(dimethylamino)-2-methoxypyridin-3-yl]-1H-indole-3-carboxylic acid (PF-249) (Figure 5B; Cokorinos et al., 2017). The binding mode was shown in Figure 5A. As shown in Figure 5B, three oxygen atoms of the native ligand can form four hydrogen bonds with the residue Arg83, Asn111, and Hie109, respectively. In addition, the benzene ring of ligand forms π-π stacking interactions with the residue Hie109 (Figure 5D). The interaction can provide the binding energy to stabilize the complex.

## Discussion

The use of AAPDs is related to a constellation of serious metabolic side effects, which may result in comorbidities such as Type 2 diabetes mellitus and cardiovascular disease, as well as contributing to the poor treatment adherence rates (Fell et al., 2008; Henderson et al., 2015). Thus, research efforts aimed at elucidating the underlying mechanisms and mitigating these AAPD-induced metabolic disturbances are of paramount importance.

Dysregulated lipid metabolism is an essential instigator for the metabolic disorder. Several animal studies showed the AAPD-induced dyslipidemia is caused by weight gain and stimulation of appetite, presumably mediated through the antagonistic effects on hypothalamic serotonin 5HT2C and histamine H1 receptors (He et al., 2014). However, the drug-induced increase of food consumption and body weight growth were only repeatedly documented in female rats (Skrede et al., 2012; He et al., 2014). As far as we know, only one study, by using high fat diet (HFD)-fed male rats, described that sustained olanzanpine treatment caused significantly elevated body weight (Fell et al., 2008). In contrast, a subsequent research showed that olanzanpine treatment led to significant reduction in body mass, both in male rats fed standard chow and HFD, which might be attributed to the drug-induced energy expenditure through enhanced brown adipose tissue thermogenesis (Fernø et al., 2015). Considering that it is very likely that the endocrine factors may influence the repeatedly verified gender specificity of metabolic effects of AAPDs and the sex hormones in female rats are fluctuant, we selected male rats in the present study. Notably, in spite of the remained elusive mechanism of gender difference, enhanced lipid biosynthesis in the periphery has consistently been reported in both male and female animals treated with CLO or olanzapine (He et al., 2014; Lian et al., 2016). The AAPDs, especially CLO and olanzapine, can induce lipid synthesis and accumulation in cultured hepatocytes and adipocytes (Lauressergues et al., 2012; Canfrán-Duque et al., 2013). Interestingly, a previous study also demonstrated that CLO induced weight loss without alteration in food intake and muscle mass in female rats, also reflecting that CLO-induced metabolic changes are independent of the weight gain and the drug may be exerting direct effects on metabolism. Our data also support this notion, showing that CLO elevated lipids concentrations in both serum and liver in the absence of increased weight gain and food consumption.

In spite of the gender and drug specific effects on the weight growth, it has been widely accepted that the AAPDs can activate SREBPs and directly induce downstream lipogenic genes in cultured cells and in both male and female animals (Fernø et al., 2015; Xuemei et al., 2015). In support of this theory, we also found that chronic CLO administration induced overexpression of both SREBP-1 and SREBP-2. SREBP-1 preferentially controls the expression of genes involved in fatty acid and triacylglycerol synthesis, while SREBP-2 is generally concerned with regulating genes involved in cholesterol production (Lauressergues et al., 2010). In accordance with the upregulated SREBPs, their target genes involved in fatty acid synthesis (ACC and FAS) and cholesterol metabolism (HMGCS, HMGCR, and LDLR) were also enhanced following prolonged CLO administration, lending more evidence to the theory that the SREBP-dependent lipid generation plays an essential role in AAPDs-induced dyslipidemia.

AMPK is a critical regulator in hepatic energy metabolism. The enzymatic activity of AMPK is dependent on the phosphorylation of Thr172 of the α-subunit (Oakhill et al., 2012). Activation of AMPK suppresses lipogenesis and leads to the inactivation of SREBPs. The present study demonstrated that CLO treatment suppressed AMPK signaling, which is in line with the previous research showing that the phosphorylation of AMPK was inhibited following CLO exposure in both primary hepatocytes and mice liver (Oh et al., 2011). Additionally, a recent study also demonstrated that olanzapine treatment can inhibit AMPK signaling and activate SREBP pathway, resulting in exacerbated adipogenesis in a 3T3-L1 cell model, whereas co-treatment with berberine, a naturally occurring alkaloid, alleviates olanzapine-induced lipid accumulation via modulating AMPK-SREBP pathway (Li et al., 2016). Likewise, the preventive effect of betahistine against olanzapine-induced dyslipidemia, which has been repeatedly documented in both animals and schizophrenia patients, is also at least partly mediated by the hepatic AMPK-SREBP system (Xuemei et al., 2015). Therefore, these data all provide evidence for the involvement of periphery AMPK-SREBP pathway in the development and treatment of AAPD-induced perturbation of lipid metabolism.

Multiple lines of evidence suggest that CUR, a polyohenol natural product, can maintain lipid homeostasis in a wide range of animal models that associated with dyslipidemia. Treatment with CUR alleviates obesity and periphery lipid accumulation, and improves insulin sensitivity in mice fed with high fat diet (Shao et al., 2012; Ding et al., 2016). CUR also prevents hyperlipidemia and hepatic fat accumulation in high-fructose-fed rats (Maithilikarpagaselvi et al., 2016). It has been recently demonstrated that CUR decreases renal lipid accumulation through AMPK-SREBP signaling in streptozotocin-induced type 1 diabetic rats (Soetikno et al., 2013). In accordance with these data, we also found that CUR partly restored CLO-induced disturbance of lipid metabolism via modulating AMPK-SREBP pathway. CUR-induced activation of AMPK was also reported in cancer cells and primary white adipocytes (Lee et al., 2009; Lone et al., 2015; Tong et al., 2016). By using molecular modeling, we further demonstrated that CUR shares common binding features with the selective AMPK allosteric ligand, PF-249 (Cokorinos et al., 2017), both of which can form hydrogen bonds with the residue Arg83 and Asn111 and form π-π stacking interactions with the residue Hie109 at the allosteric regulatory site of AMPK (PDB: 5T5T). Given the agonist effect of CUR on AMPK found in this study and previous *in vivo* and *in vitro* researches, our data further demonstrated that CUR can directly interact with AMPK and thereby regulate the SREBP-dependent lipid production. It should be noted that although CUR mitigated CLO-induced metabolic disturbance, it had no effect on the body weight gain. This scenario might be attributed to the divergent mechanisms of CLO on body weight growth and lipid metabolism. The drug-induced dyslipidemia might be due to hepatic AMPK-SREBP pathway which can be regulated by CUR, whereas CLO-induced weight loss in male rats is probably because the sedative effect of CLO that interferes with ingestion or its effect on energy expenditure in which CUR may have no role.

Collectively, in the present study, we showed for the first time that CUR successfully rescued CLO-induced dyslipidemia, indicating that adjunctive treatment of CUR is promising in the prevention against AAPD-induced lipid disturbance. Our data also provide novel evidence for the involvement of AMPK-SREBP pathway in the AAPD-induced hyperlipidemia and the beneficial effects of CUR on lipid profile. Additionally, the results from the molecular docking analysis firstly demonstrated that CUR might be a novel AMPK agonist that can directly bind to AMPK, shedding an additional interesting light on the complex pharmacological effects of CUR.

## Author contributions

ZL, PJ, and QF: Designed the study and wrote the protocol. ZL, CC, PX, RD, HC, and DL: Performed the experiments and analyzed the data. XY and DL: Did the molecular docking analysis. ZL and MY: Drafted the manuscript. PJ, CC, and QF: Revised the manuscript content. All authors read and approved the final manuscript.

### Conflict of interest statement

The authors declare that the research was conducted in the absence of any commercial or financial relationships that could be construed as a potential conflict of interest.
